# Intratympanic steroid injection and hyperbaric oxygen therapy for the treatment of refractory sudden hearing loss^☆^

**DOI:** 10.1016/j.bjorl.2016.10.013

**Published:** 2016-11-22

**Authors:** Filiz Gülüstan, Zahide Mine Yazıcı, Wesam M.E. Alakhras, Omer Erdur, Harun Acipayam, Levent Kufeciler, Fatma Tulin Kayhan

**Affiliations:** a Bakirkoy Dr Sadi Konuk Training and Research Hospital, Otolaryngology Clinic, Istanbul, Turkey; b Selcuk University, Otolaryngology Department, Konya, Turkey

**Keywords:** Salvage treatment, Intratympanic treatment, Hyperbaric oxygen therapy, Sudden hearing loss, Terapia de resgate, Tratamento intratimpânico, Oxigenoterapia hiperbárica, Perda auditiva súbita

## Abstract

**Introduction:**

Controversy surrounds the use of salvage therapies to treat sudden sensorineural hearing loss (SSNHL), with no consensus on recommendations. While several studies have demonstrated the effectiveness of intratympanic administration of steroids (ITS) and hyperbaric oxygen (HBO) treatment, few have compared the efficacy of ITS and HBO therapy in patients with refractory SSNHL.

**Objective:**

We evaluated the efficiency of ITS and HBO therapy in patients with refractory SSNHL.

**Methods:**

Patients who did not adequately benefit from systemic treatment were evaluated retrospectively. Refractory patients were defined as those who gained less than 20 dB in hearing after initial treatment. All refractory patients were informed about salvage therapy options: ITS or HBO therapy, the advantages and disadvantages of which were explained briefly. ITS involved 4 mg/mL dexamethasone administered through a 25 gauge needle. Patients underwent HBO therapy in a hyperbaric chamber where they breathed 100% oxygen for 120 min at 2.5 atmospheric pressure. The hearing levels of both groups were evaluated before the salvage therapy and at 3 months after treatment. Improvements in hearing were evaluated according to the Furahashi criteria. We also compared the two therapies in terms of speech discrimination scores (SDSs) and the recovery of all frequencies.

**Results:**

The salvage therapies generated similar results. Changes in pure tone averages and SDSs were similar for ITS and HBO therapy (*p* = 0.364 and *p* = 0.113). Comparison of SDSs and hearing thresholds at all frequencies showed similar levels of improvement.

**Conclusion:**

ITS and HBO therapy produced similar improvements in SSNHL patients, but the sample size was too small to draw definitive conclusions. Further randomized controlled studies are needed to identify the best therapy for patients with refractory sudden hearing loss.

## Resumo

**Introdução:**

Há muita controvérsia envolvendo o uso de terapias de resgate para tratar a perda auditiva neurossensorial súbita (PANSS), sem consenso sobre as recomendações. Embora vários estudos tenham demonstrado a eficácia do uso de corticoides intratimpânicos (CIT) e o tratamento com oxigenoterapia hiperbárica (HBO), poucos têm comparado a eficácia da terapia ITS e HBO em pacientes com PANSS refratária.

**Objetivo:**

Avaliamos a eficiência da terapia com CIT e HBO em pacientes com PANSS refratária.

**Método:**

Pacientes que não se beneficiaram adequadamente do tratamento sistêmico foram avaliados retrospectivamente. Pacientes refratários foram definidos como aqueles que ganharam menos de 20 dB na audição após o tratamento inicial. Todos os pacientes refratários foram informados sobre as opções de terapia de resgate: terapia com CIT ou HBO, cujas vantagens e desvantagens foram explicadas brevemente. O CIT envolveu 4 mg/mL de dexametasona administrada através de uma agulha de calibre 25. Os pacientes foram submetidos à terapia HBO em uma câmara hiperbárica onde respiraram 100% de oxigênio por 120 min a 2,5 pressão atmosférica. Os níveis de audição de ambos os grupos foram avaliados antes da terapia de resgate e três meses após o tratamento. As melhorias na audição foram avaliadas de acordo com os critérios de Furahashi. Também comparamos as duas terapias em termos de Escores de Discriminação de Fala (EDF) e a recuperação de todas as frequências.

**Resultados:**

As terapias de resgate demonstraram resultados semelhantes. As alterações nas médias de tons puros e nas EDF foram semelhantes para a terapia com CIT e HBO (*p* = 0,364 e *p* = 0,113). A comparação dos EDF e dos limiares de audição em todas as frequências mostrou níveis de melhora semelhantes.

**Conclusão:**

CIT e HBO produziram melhorias semelhantes nos pacientes com PANSS, mas o tamanho da amostra era muito pequeno para tirarmos conclusões definitivas. Estudos randomizados e controlados adicionais são necessários para identificar a melhor terapia para pacientes com perda auditiva repentina refratária.

## Introduction

The widely accepted definition of sudden sensorineural hearing loss (SSNHL) is loss of 30 dB or more for at least 3 days at three consecutive frequencies. This constitutes an otologic emergency that requires urgent treatment. The most common suggested etiologies are perilymphatic fistulas, viral infections, vascular insufficiency, and autoimmune pathologies.^1^ Systemic steroids are the most widely accepted and effective drugs for treatment of the condition.^2^ Steroids can be used orally, intravenously, or via the local intratympanic route, particularly in combination with other drugs. With steroid therapy, recovery rates increase from 32–65% to 49–89%.^3^ However, after the initial systemic treatment, approximately 30–50% of patients do not show an adequate response.^4^ For these patients, salvage therapies offer a treatment alternative.

Intratympanic administration of steroids (ITS) achieves higher perilymphatic levels compared to the systemic route.^5^ It also prevents systemic side effects, allows a higher concentration of steroids in the perilymph, and is particularly beneficial in patients who are contraindicated for systemic steroids. Therefore, it is becoming one of the most recommended treatment options for patients with SSNHL.^6^ ITS can be used as a primary treatment, salvage treatment, or in combination with systemic steroids. Its efficacy has been demonstrated.^7^, ^8^

Hyperbaric oxygen (HBO) therapy has been used to treat SSNHL since the late 1970s. It is recommended when hypoxia is thought to be the initial cause of SSNHL because it increases blood oxygen levels in the blood, thereby also increasing levels in the perilymph via diffusion.^9^ Recent studies have demonstrated that HBO therapy is effective for SSNHL patients as a salvage treatment.^10^, ^11^, ^12^

Controversy still surrounds salvage therapies in SSNHL with no consensus of the best treatment option. Several studies have demonstrated the effectiveness of ITS and HBO treatment but few studies have compared the efficacy of ITS steroids and HBO therapy for refractory SSNHL. This was the aim of the current study.

## Methods

The medical records of patients hospitalized due to SSNHL between March 2013 and August 2015 were evaluated retrospectively. SSNHL was defined as a minimum of 30 dB hearing loss at three contiguous frequencies that persisted for at least 3 days. All patients initially received intravenous methylprednisolone (250 mg) on the first day, followed by oral methylprednisolone at 1 mg/kg, which was slowly tapered over 2 weeks. Refractory SSNHL cases were defined as those that showed either no response or an improvement in PTA of less than 20 dB at the end of the second week of initial treatment.

All refractory SSNHL patients were briefly informed about the disadvantages and advantages of the procedures and all provided written informed consent. The study protocol was approved by the local ethics committee (approval number: 2015/07/10). We excluded SSHL cases with an identified etiologic factor, including previous otologic disease or operation, retro-cochlear lesions diagnosed by magnetic resonance imaging, any infectious or autoimmune diseases, and subjects who presented for primary therapy ≥30 days after onset of hearing loss. Patients who had bilateral SSNHL and those under age 18 years were also excluded. All patients were informed about salvage therapy and all agreed to undergo a second-line treatment, either ITS injection or HBO therapy. The advantages, disadvantages, and complications of the treatments were explained prior to the patient choosing their desired modality. ITS was administered three times weekly for 3 weeks, a total of nine doses, under local anesthesia with 10% lidocaine solution (Xylocaine^®^; Astra-Zeneca, Cheshire, United Kingdom) administered via a cotton ball placed within the external auditory canal for 10 min. Then, approximately 1 mL 4 mg/mL dexamethasone (Dekort; Deva Co, Istanbul, Turkey) was injected into the posterior–inferior quadrant of the tympanic membrane using a 25 gauge needle.^13^ The patient's head was then tilted about 40° toward the healthy side for 20–30 min; the patient was advised to avoid moving, speaking, swallowing, or coughing during this period. HBO therapy, chosen by 27 patients, consisted of 21 sessions administered once a day over 3 weeks. Patients breathed 100% oxygen for 120 min at 2.5 atmospheric pressure in a hyperbaric chamber. To balance middle ear pressure, the patients were told to swallow if they felt any ear discomfort.

A single audiologist used an Inter-acoustics AC40 clinical audiometer for audiologic assessment. The hearing level on the 15th day of systemic treatment was used as the initial audiometric value with the final audiometric value was measured 2 months after treatment. Improvement in PTA was evaluated according to the criteria used by Furahashi,^14^ which classifies the outcome as complete recovery, marked recovery, partial recovery, or non-recovery (Table 1). The improvement at frequencies of 250, 500, 1000, 2000, 4000, and 8000 Hz and speech discrimination (SD) were also compared before and after the treatment.

**Table 1:** Criteria used for audiologic improvement defined by Furahashi.^14^

Complete recovery	PTA 25 ≤ dB or identical to the contralateral, non-affected ear
Marked improvement	PTA improvement > 30 dB
Slight improvement	PTA improvement between 10 and 30 dB
Non-recovery	PTA improvement < 10 dB

PTA, four frequency pure-tone average (500, 1000, 2000, 4000 Hz).

The Number Cruncher Statistical System (NCSS) 2007 (Kaysville, UT, USA) was used for statistical analysis. Student's *t* test was used for descriptive statistics (mean, standard deviation, median, frequency, rate, minimum, maximum) as well as to compare quantitative data in the two group comparisons of parameters showing normal distributions. The Mann–Whitney *U* test was used to compare initial and final audiometric PTAs and values for each frequency and for the two-group comparisons of parameters in abnormal distribution. Pearson's Chi-square test and Fisher's exact test were used to compare qualitative data. Statistical significance was indicated by *p* < 0.05.

## Results

In total, 57 refractory SSNHL patients with a mean age of 42.05 ± 14.95 years (range 18–67 years) were enrolled in this study. In all, 30 patients (52.6%) were treated with ITS and the remaining 27 patients (47.4%) were treated with HBO therapy; 49.1% (*n* = 28) were women and 50.9% (*n* = 29) were men. Descriptive characteristics of the two groups are shown in Table 2. There were no significant differences in age, sex, time at which treatment began, or affected side (*p* > 0.05).

**Table 2:** Descriptive characteristics of the groups.

	Salvage therapy		*p*
	HBO	ITS	
	Mean ± SD	Mean ± SD	
*Age (years)*	45.74 ± 14.87	38.73 ± 14.46	0.077^a^
*The interval from onset to therapy*			
Days	11.33 ± 6.44	9.50 ± 4.70	0.466^b^
Median	8	8	

	Salvage therapy		*p*
	HBO	ITS	
	*n* (%)	*n* (%)	
*Gender*			
Female	16 (59.3)	12 (40.0)	0.146^c^
Male	11 (40.7)	18 (60.0)	

*Ear side*			
Right	11 (59.3)	12 (40.0)	0.146^c^
Left	16 (40.7)	18 (60.0)	

*Chronic disease*			
No	22 (81.5)	28 (93.3)	0.238^d^
Yes	5 (18.5)	2 (6.7)	

ITS, intratympanic steroid; HBO, hyperbaric oxygen; SD, standard deviation.

a Student *t* test.

b Mann–Whitney *U* test.

c Pearson's Chi-square test.

d Fisher's Exact test.

Before salvage treatment, the average PTAs were 71.47 ± 25.32 and 60.59 ± 22.75 in the ITS and HBO group, respectively, and there were no statistically significant differences between these values (*p* > 0.05). After treatment, the averages decreased to 51.27 ± 30.76 for ITS and 47.78 ± 24.43 for HBO therapy. The hearing gains were 20.20 ± 19.77 and 12.81 ± 13.31 in the ITS group and HBO group, respectively. There were no significant differences between the post-treatment values and gains (*p* = 0.217). The changes in SDSs were 16.13 ± 22.76 and 8.59 ± 16.14 in the ITS and HBO groups, respectively, and there were no statistically significant differences between the two groups (*p* = 0.113).

Complete recovery was observed in 12 (21.1%) patients. Twenty-five (43.9%) patients were resistant to salvage therapy, and were defined as the non-recovery group. Partial improvement was noticed in 12 patients (21.1%) and marked improvement was noticed in 8 patients (14%). When the groups were evaluated in terms of full improvement, marked improvement, or partial improvement according to the criteria of Furahashi, no significant differences were detected between the two salvage treatment groups (*p* > 0.05) (Table 3).

**Table 3:** Hearing recoveries of the groups.

	Salvage therapies		*p*
	HBO	ITS	
	*n* (%)	*n* (%)	
*Full recovery*			0.837
No	21 (77.8)	24 (80.0)	
Yes	6 (22.2)	6 (20.0)	

*Recovery degree*			0.364
No	13 (48.1)	12 (40.0)	
Slight	7 (25.9)	5 (16.7)	
Marked	7 (25.9)	13 (43.3)	

*Recovery degree*			0.536
No	13 (48.1)	12 (40.0)	
Slight + marked	14 (51.9)	18 (60.0)	

Pearson's Chi-square test.

ITS, intratympanic steroid; HBO, hyperbaric oxygen.

## Discussion

In the majority of SSNHL patients, the specific cause of the condition is unknown and these cases are referred to as idiopathic. As the exact etiopathogenesis is not well understood, the treatment of SSNHL remains controversial. Alone or in combination with other treatments, systemic steroids are the most commonly accepted treatment for SSNHL.^2^ Additional therapies are needed for the patients who do not adequately improve with initial systemic steroid treatment. Most authors support the beneficial effects of salvage therapies and favor them over a second course of systemic steroid treatment, because ITS and HBO therapy are associated with fewer adverse effects.

The advantages of ITS are its decreased systemic side effects compared to systemic steroid administration and its ability to deliver a higher concentration of steroids to the perilymph.^5^ Salvage ITS therapy has been shown to provide an additional hearing gain in about 38–53% of patients.^6^, ^15^ The American Academy of Otolaryngology recommends ITS for patients who exhibit incomplete recovery. In addition, meta-analyses that investigated the efficacy of ITS as a salvage treatment demonstrated significant reduction in hearing thresholds.^6^, ^7^, ^8^, ^9^, ^10^, ^11^, ^12^, ^13^, ^14^, ^15^, ^16^, ^17^, ^18^

In our clinic, salvage therapy is routinely recommended for refractory SSNHL patients, almost all of them opt to undergo a second line treatment. Both salvage therapies were explained to all patients, 30 of them chose ITS therapy. The average PTA of the ITS group was 71.47 ± 25.32 before the treatment and 51.27 ± 30.76 after treatment. The mean gain was 20.20 ± 19.77 and the change in SDSs was 16.13 ± 22.76.

Some authors have suggested that ischemia is an important pathologic factor in the development of SSNHL, and HBO therapy is thought to be useful for these patients.^10^, ^11^, ^12^ Nagahara et al.^19^ demonstrated that perilymphatic oxygen tension is significantly decreased in SSNHL patients compared to controls. Animal studies have demonstrated that oxygen can easily diffuse across the membranes of the inner ear from the blood, and that the partial pressure of oxygen in the perilymph of the inner ear increases during HBO therapy.^9^ This increased oxygen concentration in the inner ear fluids may nourish sensorial elements of the cochlea.^20^

Many studies have found HBO therapy to be effective.^20^, ^21^, ^22^ In recent years, some authors have also found HBO therapy successful as a salvage therapy.^10^, ^11^, ^12^, ^23^, ^24^, ^25^ Lamm et al.^26^ reported a meta-analysis of HBO therapy as a salvage therapy and reported a hearing gain (>10 dB) in 86% of patients. In our study, 27 patients agreed to undergo HBO therapy. The average PTA of the HBO group was 60.59 ± 22.75 before the treatment and 47.78 ± 24.43 afterwards. The mean gain was 12.81 ± 13.31 and the change in SDSs was 8.59 ± 16.14.

Few studies have evaluated the efficiency of ITS and HBO therapy for refractory SSNHL patients. Yang et al.^27^ recently compared salvage therapy using HBO and ITS treatments and found that neither modality was superior. Cvoronic et al.^28^ compared HBO therapy and ITS for salvage treatment in a randomized prospective study and found that both options were successful. In our study of refractory SSNHL patients, we compared the hearing gain and SDS results of these two salvage therapies, which produced similar hearing gains and SDSs. ITS therapy showed better healing in terms of both hearing gains and SDSs compared to HBO therapy but there were no statistically significant differences between the groups.

There were several limitations to this study. First, it lacked randomization and a control group, given its retrospective design. Only five patients declined the salvage treatment, which yielded a small control group, insufficient to allow effective comparisons. However, the main aim of this study was to compare ITS and HBO therapy as salvage treatments.

ITS and HBO therapy have different mechanisms: ITS reduces inflammation in the inner ear by diffusion via the round window while HBO therapy increases oxygen concentration in the inner ear by diffusion from the blood, thereby aiding the recovery of affected sensorial elements of the cochlea. In our study, ITS treatment yielded better hearing gains than did HBO therapy, but further larger studies with randomized controlled groups are needed to identify the best treatment for refractory SSNHL patients.

## Conclusion

ITS and HBO therapy showed similar results in SSNHL patients, but further randomized controlled studies are needed to demonstrate the best therapy for patients with refractory sudden hearing loss.

## Conflicts of interest

The authors declare no conflicts of interest.

## References

[1] Levie P., Desgain O., de Burbure C., Germonpré P., Monnoye J.P., Thill M.P. (2007). Sudden hearing loss. B-ENT.

[2] Wilson W.R., Byl F.M., Laird L.N. (1980). The efficiency of steroids in the treatment of idiopathic sudden hearing loss. A double-blind clinical study. Arch Otolaryngol.

[3] Moskowitz D., Lee K.J., Smith H.W. (1984). Steroid use in idiopathic sudden sensorineural hearing loss. Laryngoscope.

[4] Plaza G., Herráiz C. (2007). Intratympanic steroids for treatment of sudden hearing loss after failure of intravenous therapy. Otolaryngol Head Neck Surg.

[5] Chandrasekhar S.S. (2001). Intratympanic dexamethasone for sudden sensorineural hearing loss: clinical and laboratory evaluation. Otol Neurotol.

[6] Ho G.M., Lin H.C., Shu M.T., Yang C.C., Tsai H.T. (2004). Effectiveness of intratympanic dexamethasone injection in sudden-deafness patients as salvage treatment. Laryngoscope.

[7] Erdur O., Kayhan F.T., Cirik A.A. (2014). Effectiveness of intratympanic dexamethasone for refractory sudden sensorineural hearing loss. Eur Arch Otorhinolaryngol.

[8] Tezer M.S., Baran Y., Erdur O., Ata N., Arslanhan M. (2013). Comparison of systemic, intratympanic and combination therapy of the steroids for the treatment of sudden sensorineural hearing loss. J Med Sci.

[9] Lamm K., Lamm C., Arnold W. (1998). Effect of isobaric oxygen versus hyperbaric oxygen on the normal and noise-damaged hypoxic and ischemic guinea pig inner ear. Adv Otorhinolaryngol.

[10] Muzzi E., Zennaro B., Visentin R., Soldano F., Sacilotto C. (2010). Hyperbaric oxygen therapy as salvage treatment for sudden sensorineural hearing loss: review of rationale and preliminary report. J Laryngol Otol.

[11] Pezzoli M., Magnano M., Maffi L., Pezzoli L., Marcato P., Orione M. (2015). Hyperbaric oxygen therapy as salvage treatment for sudden sensorineural hearing loss: a prospective controlled study. Eur Arch Otorhinolaryngol.

[12] Psillas G., Ouzounidou S., Stefanidou S., Kotsiou M., Giaglis G.D., Vital I. (2015). Hyperbaric oxygen as salvage treatment for idiopathic sudden sensorineural hearing loss. B-ENT.

[13] Sevil E., Bercin S., Muderris T., Gul F., Kiris M. (2015). Comparison of two different steroid treatments with hyperbaric oxygen for idiopathic sudden sensorineural hearing loss. Eur Arch Otorhinolaryngol.

[14] Furuhashi A., Matsuda K., Asahi K., Nakashima T. (2002). Sudden deafness: longterm follow-up and recurrence. Clin Otolaryngol.

[15] Xenellis J., Papadimitriou N., Nikolopoulos T., Maragoudakis P., Segas J., Tzagaroulakis A. (2006). Intratympanic steroid treatment in idiopathic sudden sensorineural hearing loss: a control study. Otolaryngol Head Neck Surg.

[16] Ng J.H., Ho R.C., Cheong C.S., Ng A., Yuen H.W., Ngo R.Y. (2015). Intratympanic steroids as a salvage treatment for sudden sensorineural hearing loss? A meta-analysis. Eur Arch Otorhinolaryngol.

[17] Li H., Feng G., Wang H., Feng Y. (2015). Intratympanic steroid therapy as a salvage treatment for sudden sensorineural hearing loss after failure of conventional therapy: a meta-analysis of randomized, controlled trials. Clin Ther.

[18] Garavello W., Galluzzi F., Gaini R.M., Zanetti D. (2012). Intratympanic steroid treatment for sudden deafness: a meta-analysis of randomized controlled trials. Otol Neurotol.

[19] Nagahara K., Fisch K., Yagi M. (1983). Perilymph oxygenation in sudden and progressive sensorineural hearing loss. Acta Otolaryngol.

[20] Fujimura T., Suzuki H., Shiomori T., Udaka T., Mori T. (2007). Hyperbaric oxygen and steroid therapy for idiopathic sudden sensorineural hearing loss. Eur Arch Otorhinolaryngol.

[21] Bennett M.H., Kertesz T., Perleth M., Yeung P. (2007).

[22] Filipo R., Attanasio G., Viccaro M., Russo F.Y., Mancini P., Rocco M. (2012). Hyperbaric oxygen therapy with short duration intratympanic steroid therapy for sudden hearing loss. Acta Otolaryngol.

[23] Ohno K., Noguchi Y., Kawashima Y., Yagishita K., Kitamura K. (2010). Secondary hyperbaric oxygen therapy for idiopathic sudden sensorineural hearing loss in the subacute and chronic phases. J Med Dent Sci.

[24] Horn C.E., Himel H.N., Selesnick S.H. (2005). Hyperbaric oxygen therapy for sudden sensorineural hearing loss: a prospective trial of patients failing steroid and antiviral treatment. Otol Neurotol.

[25] Desloovere C., Knecht R., Germonpré P. (2006). Hyperbaric oxygen therapy after failure of conventional therapy for sudden deafness. B-ENT.

[26] Lamm K., Lamm H., Arnold W. (1998). Effect of hyperbaric oxygen therapy in comparison to conventional or placebo therapy or no treatment in idiopathic sudden hearing loss, acoustic trauma, noise-induced hearing loss and tinnitus. A literature survey. Adv Otorhinolaryngol.

[27] Yang C.H., Wu R.W., Hwang C.F. (2013). Comparison of intratympanic steroid injection, hyperbaric oxygen and combination therapy in refractory sudden sensorineural hearing loss. Otol Neurotol.

[28] Cvorovic L., Jovanovic M.B., Milutinovic Z., Arsovic N., Djeric D. (2013). Randomized prospective trial of hyperbaric oxygen therapy and intratympanic steroid injection as salvage treatment of sudden sensorineural hearing loss. Otol Neurotol.

